# Perforated Type III Amyand's Hernia With Retroperitoneal and Scrotal Abscesses and Testicular Ischemia Requiring Orchiectomy: A Case Report

**DOI:** 10.7759/cureus.115496

**Published:** 2026-08-31

**Authors:** Sergio Miguel Suárez Fuentes, Mauro A Cardenas, Ricardo Y Topete De Robles, Martín Rodríguez-Garza, Valeria Gomez Marquez

**Affiliations:** 1 Surgery, Hospital Regional Valentín Gómez Farías, Instituto de Seguridad y Servicios Sociales de los Trabajadores del Estado (ISSSTE), Zapopan, MEX; 2 Surgery, Hospital General ISSSTE Presidente General Lázaro Cárdenas del Río, Chihuahua, MEX; 3 Surgery, Hospital Civil de Tepic, Tepic, MEX; 4 General Surgery, Hospital Regional, Instituto de Seguridad y Servicios Sociales de los Trabajadores del Estado (ISSSTE), Monterrey, MEX

**Keywords:** amyand's hernia, case report, inguinal hernia, orchiectomy, perforated appendicitis, retroperitoneal abscess, scrotal abscess, testicular ischemia

## Abstract

Amyand's hernia is defined as the presence of the vermiform appendix within an inguinal hernia and becomes particularly challenging when appendicitis progresses to perforation and extra-abdominal sepsis. We report a 60-year-old man with one week of progressive abdominal pain, reduced appetite, constipation, and impaired ambulation followed by three days of painful right inguinoscrotal enlargement. Examination showed an irreducible, inflamed right inguinoscrotal mass. Laboratory testing demonstrated leukocytes of 19.35 × 10³/µL, neutrophils of 92%, and bands of 13%. Ultrasonography showed a right inguinal wall defect with non-reducing omentum and bowel and complex scrotal contents. Emergency inguinal exploration drained approximately 400 mL of frank purulent material and demonstrated severe ischemic changes of the right spermatic cord and testis. Given the extent of the operative findings, midline laparotomy was performed; a retroperitoneal abscess communicating with the inguinal region and a gangrenous perforated appendix were identified, with the perforation approximately 1.5 cm from the appendiceal base. Appendectomy, retroperitoneal and scrotal drainage, Penrose drain placement, right orchiectomy for nonviability, and Shouldice tissue repair without mesh were performed. Recovery was favorable without known immediate complications. This case illustrates a severe Type III Amyand's hernia with simultaneous retroperitoneal and inguinoscrotal septic extension and irreversible testicular ischemia. In grossly contaminated presentations, prompt source control, excision of nonviable tissue, and nonprosthetic hernia repair remain pragmatic priorities.

## Introduction

Amyand's hernia denotes the presence of the vermiform appendix within an inguinal hernia sac [1-3]. Although the entity itself is uncommon, clinically important disease arises when the herniated appendix becomes inflamed, incarcerated, gangrenous, or perforated. In the 20-year systematic review by Manatakis et al., 231 publications described 442 patients; most acute presentations were initially treated for complications of the hernia rather than for a recognized Amyand's hernia, underscoring the persistent preoperative diagnostic challenge [1]. Systematic reviews and contemporary reviews likewise emphasize the heterogeneity of presentation and the absence of high-level evidence for a single operative strategy [2,3].

Because operative management is closely linked to appendiceal pathology and the extent of sepsis, Losanoff and Basson proposed a four-tier classification [4]. Type I describes a normal appendix within an inguinal hernia; Type II, acute appendicitis confined to the hernia without abdominal wall or peritoneal sepsis; Type III, acute appendicitis accompanied by peritonitis or abdominal wall sepsis; and Type IV, Amyand's hernia associated with additional abdominal pathology [4]. The present case corresponds to Type III disease. Although this classification provides a useful framework, evidence regarding complex presentations with extensive extra-sac septic spread remains limited and is derived predominantly from case reports and small series [1,5,6].

Scrotal abscess, spermatic cord necrosis, Fournier-type extension, and loss of testicular viability are among the rarest reported complications of perforated Amyand's hernia [7-11]. Reports describing the simultaneous occurrence of retroperitoneal and inguinoscrotal septic extension with irreversible testicular ischemia are particularly limited. The present case is notable for the combined presence of a gangrenous perforated appendix, a retroperitoneal abscess communicating with the inguinal region, an approximately 400-mL scrotal abscess, and irreversible right testicular ischemia requiring orchiectomy. We report the diagnostic and operative course, emphasizing this unusual pattern of septic spread and its implications for intraoperative decision-making. This report was prepared in accordance with the SCARE 2025 guideline [12].

## Case presentation

A 60-year-old man with no reported relevant comorbidities or previous surgical history presented to the emergency department after one week of intense, progressive abdominal pain accompanied by anorexia, inability to evacuate, and difficulty ambulating. Three days before admission, he developed progressive, painful enlargement of the right inguinal and scrotal regions. Examination demonstrated a nonreducible right inguinoscrotal mass with erythema, marked swelling, and tenderness. The working diagnosis was a complicated incarcerated right inguinal hernia, and urgent operative exploration was planned.

Initial laboratory evaluation demonstrated leukocytosis with marked neutrophilia and bandemia, whereas hemoglobin was within the expected adult male reference range. The available admission hematologic results are summarized in Table 1.

**Table 1:** Initial hematologic laboratory findings at emergency evaluation.

Test	Result	Reference range*	Interpretation
White blood cell count	19.35 × 10³/µL	4.5-11.0 × 10³/µL	High
Hemoglobin	14.20 g/dL	13-18 g/dL (adult male)	Within range
Neutrophils	92%	40%-60%	High
Bands (young neutrophils)	13%	0-3%	High

Inguinoscrotal ultrasonography demonstrated echogenic scrotal contents without posterior acoustic shadowing and no Doppler flow within those contents. The inguinal canal showed a wall defect consistent with an inguinal hernia, with protrusion of omentum and a bowel loop that did not reduce during the study (Figure 1).

**Figure 1:**
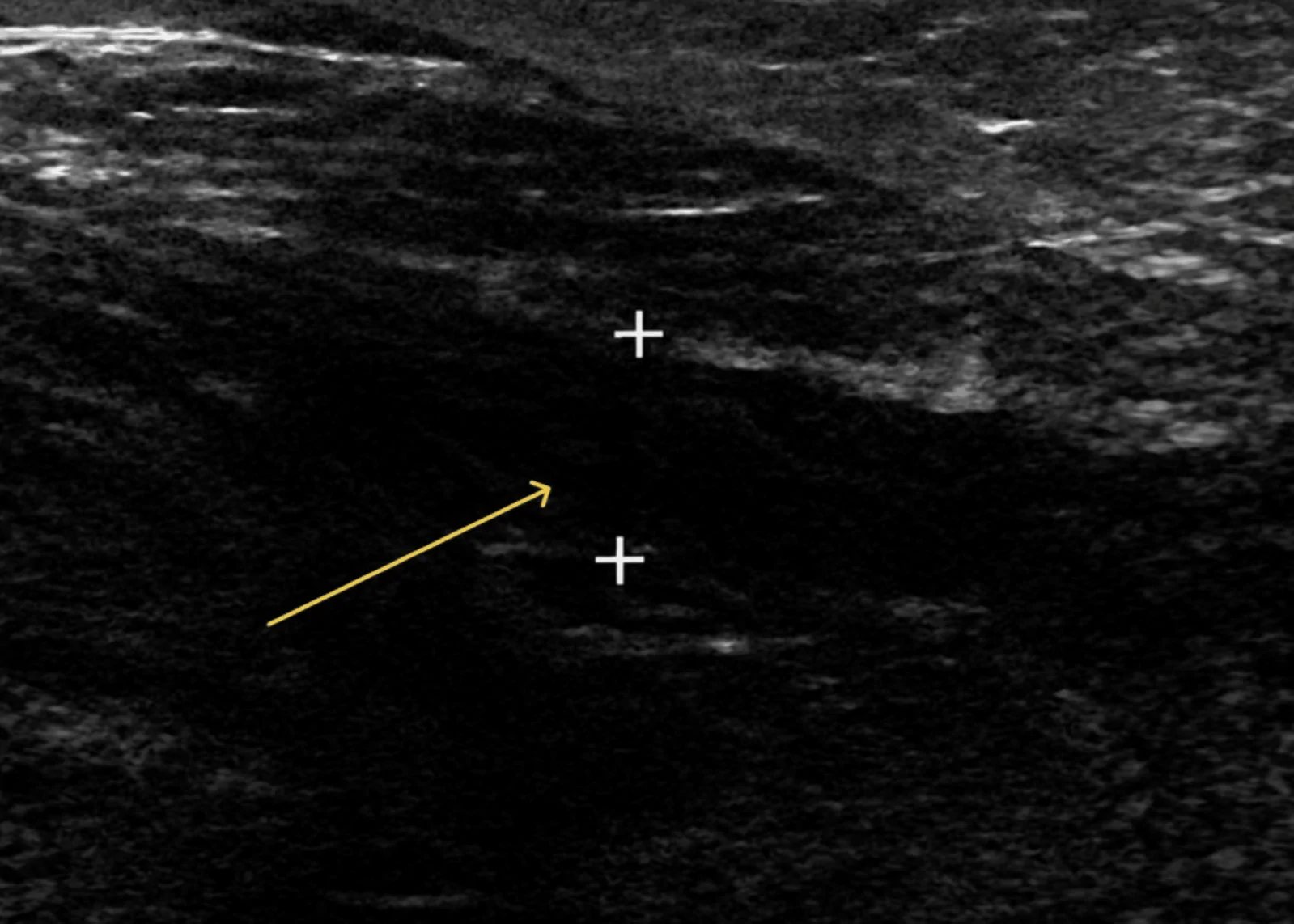
Preoperative right inguinoscrotal ultrasonography. Defect in the right inguinal canal compatible with an inguinal hernia. The “+” symbols represent the electronic ultrasound calipers used to delineate and measure the hernia defect.

The appendix was not initially identified in its expected position. The cecum was mobilized, revealing a retroperitoneal abscess that communicated toward the inguinal region. After inflammatory adhesions were released, a gangrenous, perforated appendix was identified; the perforation was approximately 1.5 cm from the appendiceal base (Figure 2). These findings established the diagnosis of a complicated Amyand's hernia with perforated appendicitis and abdominal wall/retroperitoneal sepsis, corresponding to Losanoff-Basson Type III disease [4].

**Figure 2:**
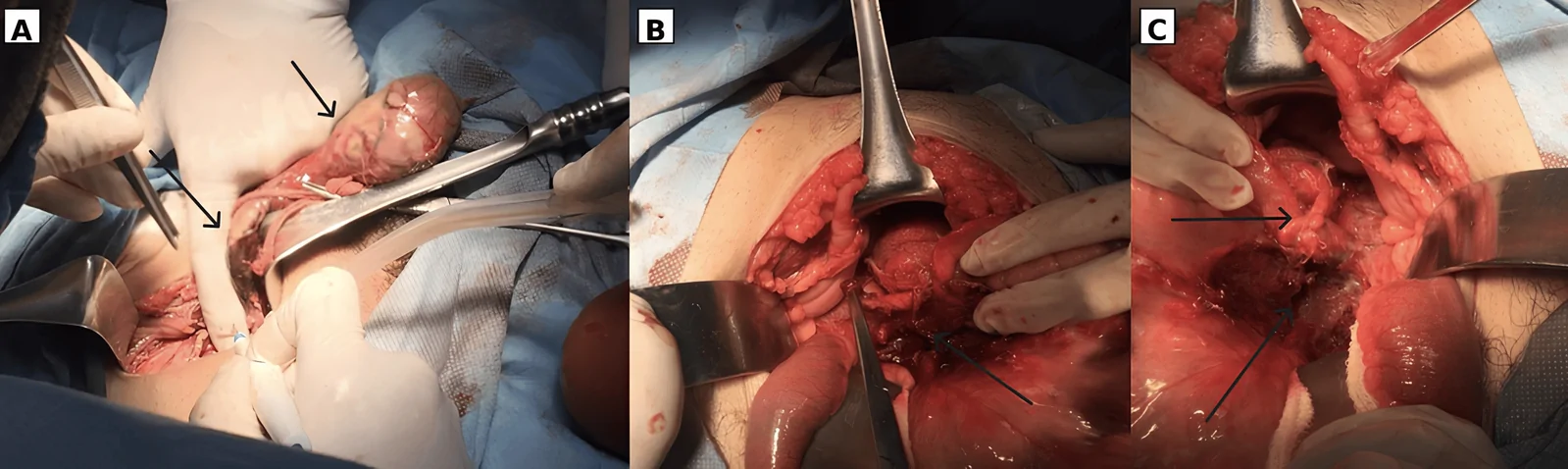
Intraoperative findings of complicated Amyand's hernia. (A) Severe congestion and ischemic changes involving the right spermatic cord and testicular parenchyma. (B) Gangrenous, perforated appendix; the perforation was approximately 1.5 cm from the appendiceal base. (C) Hernia defect and gangrenous appendix during abdominal exploration.

Appendectomy was performed, followed by drainage and irrigation of the retroperitoneal and inguinoscrotal infected compartments. A Penrose drain was placed. Urology was consulted intraoperatively because of the severe ischemic changes; the right testis was judged nonviable, and right orchiectomy was performed. The inguinal defect was repaired using the Shouldice technique, a nonprosthetic multilayer tissue repair of the posterior inguinal wall, because the operative field was grossly contaminated. The operation concluded without reported intraoperative complications.

The postoperative recovery was favorable, with clinical control of the septic process and no known immediate complications. The patient was subsequently discharged in clinically favorable condition. Histopathological examination of the appendectomy specimen confirmed a necrotic vermiform appendix, consistent with the intraoperative finding of gangrenous appendicitis.

## Discussion

Although Amyand's hernia has been described for centuries, modern evidence remains dominated by case reports and small series. The 442-patient systematic review by Manatakis et al. demonstrated that acute cases most often present as complications of the groin hernia rather than as recognizable appendicitis, and preoperative identification remains uncommon [1]. Papaconstantinou et al. and Michalinos et al. reached similar conclusions: clinical findings overlap substantially with incarcerated or strangulated inguinal hernia, and definitive diagnosis is frequently intraoperative [2,3]. Our patient followed this pattern: the irreducible, inflamed inguinoscrotal mass appropriately triggered urgent exploration but did not identify the appendix as the source before surgery.

The pathophysiology of appendicitis within an Amyand's hernia is not fully established. Proposed mechanisms include adhesional fixation, repeated trauma, and compression of the appendix or mesoappendix at the hernia neck, producing venous and lymphatic obstruction followed by edema, ischemia, bacterial proliferation, and eventual gangrene or perforation [3,6]. Once perforation occurs, the hernia sac may initially contain contamination while pus dissects along contiguous tissue planes within the groin and scrotum. In our case, the retroperitoneal abscess communicating toward the inguinal region supports contiguous septic extension, although the precise anatomical route cannot be reconstructed retrospectively.

Cross-sectional imaging can establish the diagnosis before surgery when the appendix can be traced from the cecum into the hernia sac and can additionally show inflammatory change, abscess, or perforation [13]. However, imaging should not delay surgery in a clinically complicated irreducible hernia with substantial local inflammation. Ultrasonography in our patient established a non-reducing inguinal hernia and abnormal scrotal contents, providing sufficient evidence of a complicated process to proceed to urgent exploration; computed tomography was not obtained. The absence of computed tomography limits preoperative anatomical characterization, but the operative indication was already clear.

The Losanoff-Basson classification was designed to translate intraoperative appendiceal pathology into a rational hernia strategy [4]. Our patient most closely fits Type III because perforated appendicitis was accompanied by sepsis beyond the hernia sac. In this setting, the major management principle is source control, followed by repair appropriate to the contamination burden. Manatakis et al. reported a strong preference for endogenous tissue repair in heavily contaminated acute cases [1], and both the 2025 Cureus systematic review and the systematic review by Bratu et al. support tissue repair or staged reconstruction when gross contamination is present [5,6]. The Shouldice repair used in this case is a mesh-free multilayer tissue reconstruction and avoided implantation of permanent prosthetic material into a field containing frank pus. Salemis et al. similarly reported appendectomy and Shouldice herniorrhaphy without mesh for a perforated appendix with periappendicular abscess [14].

The combined inguinal and abdominal approach was dictated by the distribution of disease. Groin exploration allowed drainage of the scrotal collection and assessment of cord and testicular viability, while midline laparotomy allowed cecal mobilization, appendectomy, and drainage of the retroperitoneal source. Orchiectomy was performed only because the right testis was irreversibly nonviable. Comparable reports describe spermatic cord necrosis requiring orchiectomy [8,9], scrotal abscess with testicular necrosis [10], and Fournier-type necrotizing extension [11]. Juan et al. reported perforated Amyand's hernia with scrotal abscess diagnosed preoperatively by computed tomography [7], whereas Oremule and Ashrafi described a periappendicular abscess managed with appendectomy, Bassini repair, and drainage [15]. Across the representative adult reports reviewed for this manuscript, we did not identify another case reproducing the complete combination observed here: a gangrenous, perforated appendix, a retroperitoneal abscess communicating with the inguinal region, an approximately 400-mL scrotal abscess, and irreversible testicular ischemia. The principal contribution of this report is therefore the documentation of this combined septic distribution rather than the mere presence of an Amyand's hernia.

This case also illustrates a limitation of classification-based algorithms. Losanoff-Basson is useful for guiding appendectomy and hernia-repair strategy, but it does not capture every consequence of extra-sac sepsis, such as retroperitoneal extension, scrotal abscess, or testicular nonviability. From a diagnostic perspective, marked purulence or septic findings disproportionate to the expected groin pathology may indicate disease extending beyond the hernia sac and warrant evaluation for an intra-abdominal or retroperitoneal source.

The principal strengths of this report are the direct operative documentation of the appendiceal, retroperitoneal, inguinoscrotal, and testicular findings and the availability of contemporaneous ultrasonographic and intraoperative images. Histopathological examination confirmed necrosis of the vermiform appendix, supporting the intraoperative diagnosis of gangrenous appendicitis. A limitation of this report is that computed tomography was not performed preoperatively; therefore, the full extent of the retroperitoneal and inguinoscrotal septic process could not be characterized radiologically before surgery. Long-term follow-up data were also unavailable. These limitations do not alter the directly observed operative anatomy or the principal educational message of the case.

## Conclusions

The key finding of this case was an unusually extensive Type III Amyand's hernia in which a gangrenous, perforated appendix produced contiguous retroperitoneal and inguinoscrotal sepsis, an approximately 400-mL scrotal abscess, and irreversible right testicular ischemia requiring orchiectomy. Clinically, in a patient with a painful, irreducible right inguinoscrotal mass whose inflammatory burden appears disproportionate to an uncomplicated incarcerated hernia, Amyand's hernia with extra-sac septic extension may warrant consideration.

In this case, adequate source control required drainage of all involved infected compartments, removal of nonviable tissue, appendectomy, and nonprosthetic hernia repair in the setting of gross contamination. The Losanoff-Basson framework remains useful for orientation; however, the findings of this case support tailoring operative management to intraoperative anatomy, contamination burden, and extent of sepsis rather than extrapolating a single operative strategy to all patients.

Future multicenter registries and systematic collection of complicated Amyand's hernia cases should evaluate predictors of extra-sac septic extension, the role of preoperative cross-sectional imaging in complex presentations, outcomes of nonprosthetic versus staged or prosthetic hernia repair in contaminated fields, and long-term recurrence and testicular outcomes.
